# Supplementary material for: A Lagrangian model for drifting ecosystems reveals heterogeneity-driven enhancement of marine plankton blooms
Source: Nat Commun. 2023 Sep 29;14:6092. doi: 10.1038/s41467-023-41469-2 (PMC10541867; doi:10.1038/s41467-023-41469-2)
Supplement: Supplementary file 1 — Supplementary Information [file 41467_2023_41469_MOESM1_ESM.pdf]

SUPPLEMENTARY INFORMATION: A Lagrangian model for drifting ecosystems reveals heterogeneity-driven enhancement of marine plankton blooms

Enrico Ser-Giacomi[1,2*]
[1] *Department of Earth, Atmospheric and Planetary Sciences, Massachusetts Institute of Technology, 54-1514 MIT, Cambridge, MA 02139, USA.*
[2] *IFISC (CSIC-UIB), Institute for Cross-Disciplinary Physics and Complex Systems, Palma de Mallorca, Spain.*

Ricardo Martinez-Garcia[3,4]
[3] *ICTP South American Institute for Fundamental Research & Institute of Theoretical Physics, Universidade Estadual Paulista - UNESP, Rua Dr.Bento Teobaldo Ferraz 271, Bloco 2 - Barra Funda, 01140-070 São Paulo,SP, Brazil*
[4] *Center for Advanced Systems Understanding (CASUS); Helmholtz-Zentrum Dresden-Rossendorf (HZDR), Görlitz, Germany.*

Stephanie Dutkiewicz[1] and Michael J. Follows[1]
[1] *Department of Earth, Atmospheric and Planetary Sciences, Massachusetts Institute of Technology, 54-1514 MIT, Cambridge, MA 02139, USA.*

[*] enrico.sergiacomi@gmail.com

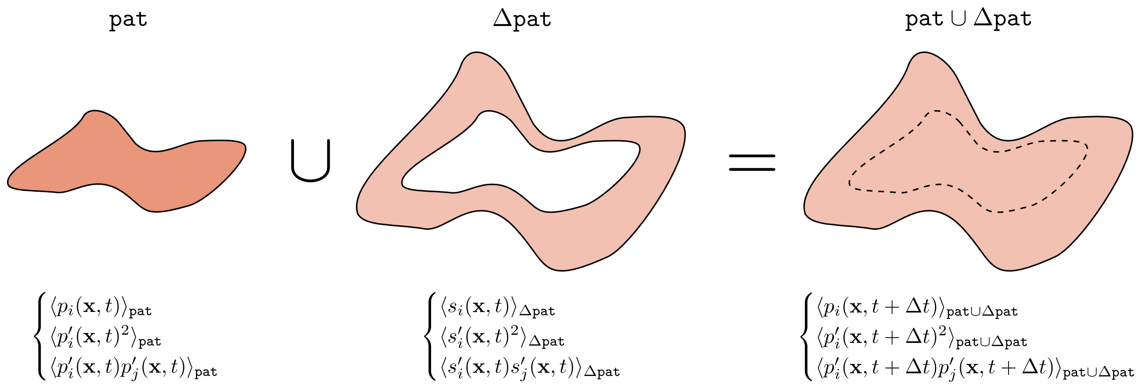

SUPPLEMENTARY FIGURE 1. Entrainment of surrounding water into a Lagrangian patch modifies its internal tracers distribution. The amount of water that enters in the patch for unit of time corresponds to the area increase rate. In a given time step Δt, the portion of the ocean surface $\Delta\mathtt{pat}$ is added to the patch region $\mathtt{pat}$. Consequently, means, variances and covariance change depending on the different proprieties of the intruded water.

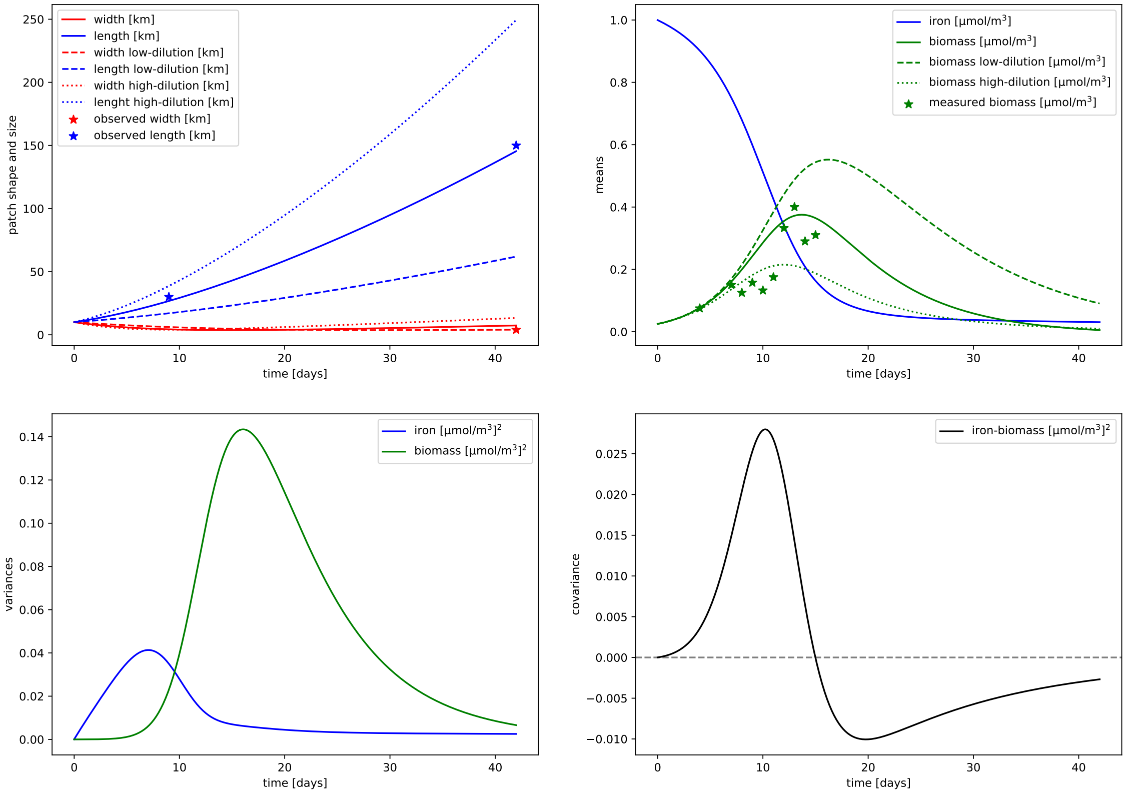

SUPPLEMENTARY FIGURE 2. Predictions of the SOIREE bloom behave when we arbitrarily increase or decrease strain and diffusion. Solid lines correspond to modeled variables when we reproduce the observed bloom dilution matching the evolution of the patch length and width (as in Fig. 5 of main text), with Spearman correlation of 0.86 (p-*value*=0.001). Dashed lines represent model predictions when we decrease dilution using $\gamma = 0.06$ and $\kappa = 0.05$, with Spearman correlation of 0.84 (p-*value*=0.002). Dotted lines represent instead model predictions when we increase dilution using $\gamma = 0.18$ and $\kappa = 0.2$, with Spearman correlation of 0.76 (p-*value*=0.009). We stress that the statistical robustness of the above analysis is however modest due to the relatively low number of data points.

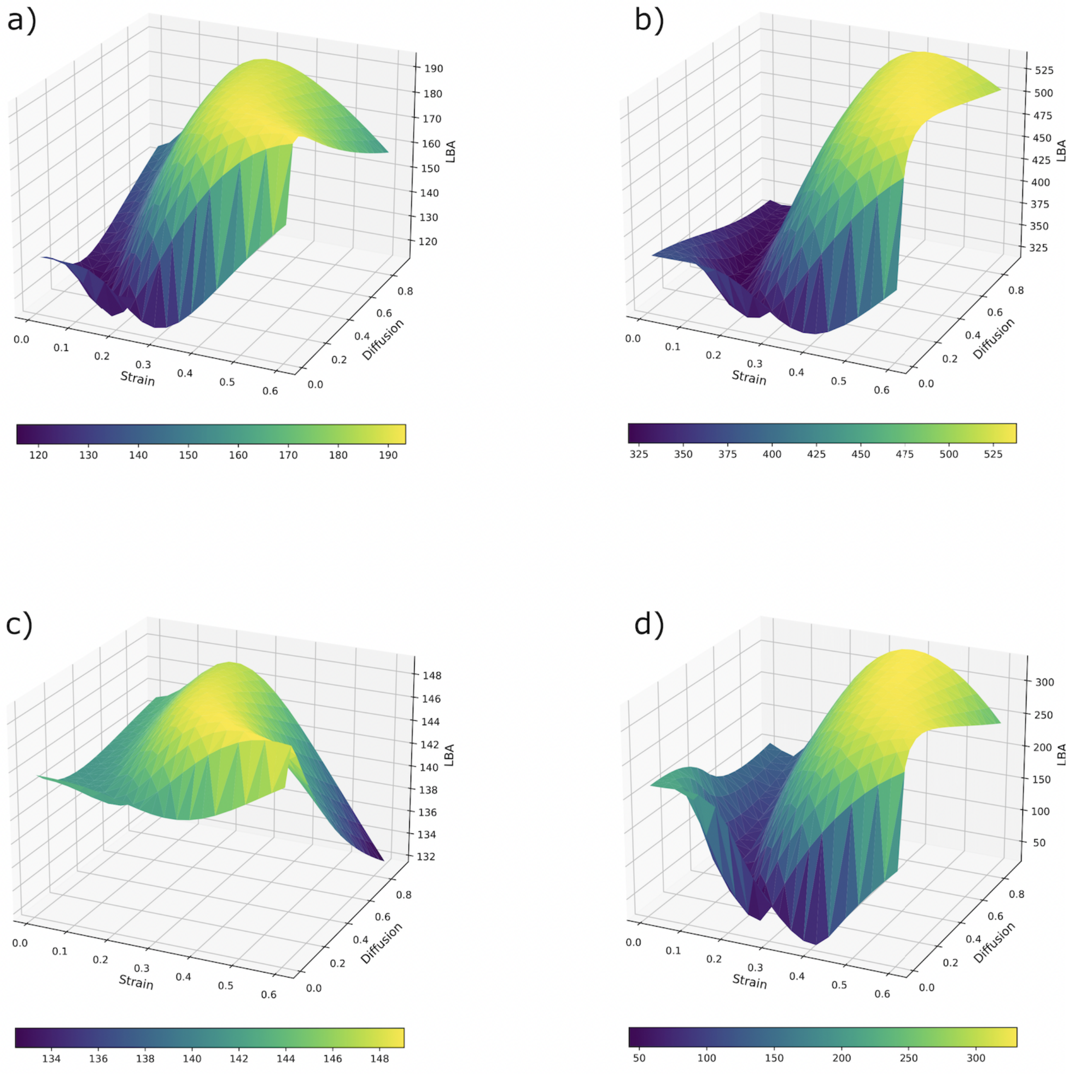

SUPPLEMENTARY FIGURE 3. Sensitivity analysis to the physical parameters of the model. We show the LBA surface of the heterogeneous ensemble for different choices of specific parameters (all the others are equal to the ones used in Fig. 6 of main text). In panel a) we use an initial patch size of $S = 7.5$ km while in panel b) we use $S = 12.5$. In panel c) we use an integration time of $\tau = 15$ days while in panel d) we use $\tau = 45$.

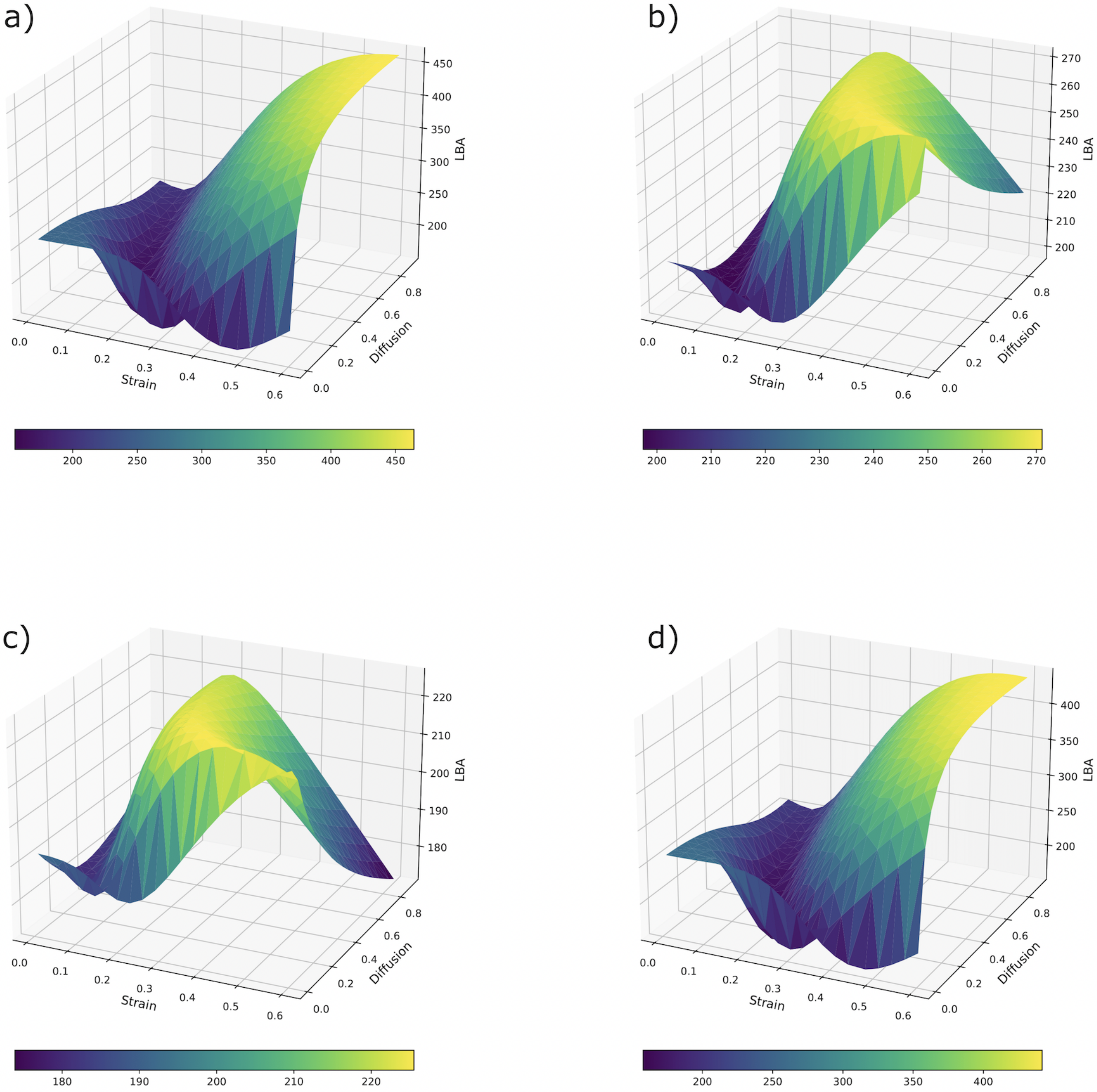

SUPPLEMENTARY FIGURE 4. Sensitivity analysis to the biological parameters of the model. We show the LBA surface of the heterogeneous ensemble for different choices of specific parameters (all the others are equal to the ones used in Fig. 6 of main text). In panel a) we use a half-saturation constant of $k = 1.5$ μmol/m^3 with a surrounding nutrient concentration of 0.08 μmol/m^3 while in panel b) we use $k = 2.5$ with a surrounding nutrient concentration of 0.128. In panel c) we use a maximum growth-rate of $\nu = 0.94$ day^{-1} with a surrounding nutrient concentration of 0.138 μmol/m^3 while in panel d) we use $\nu = 1.56$ with a surrounding nutrient concentration of 0.081 μmol/m^3.

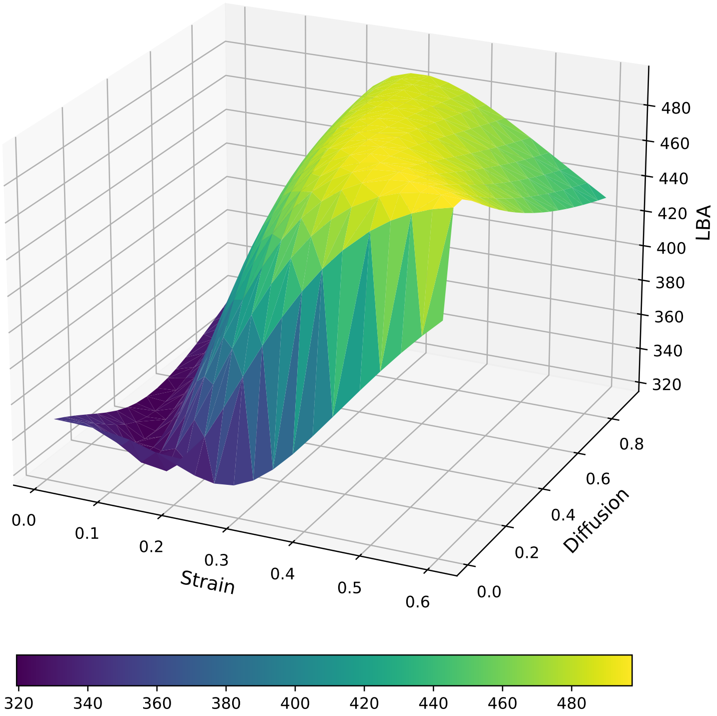

SUPPLEMENTARY FIGURE 5. Sensitivity analysis to the recycling of resource. We show the model the LBA surface of the heterogeneous ensemble when we implement a complete remineralization dynamics, that means setting $\alpha = 1$. All other parameters are equal to the ones used in Fig. 6 of main text.

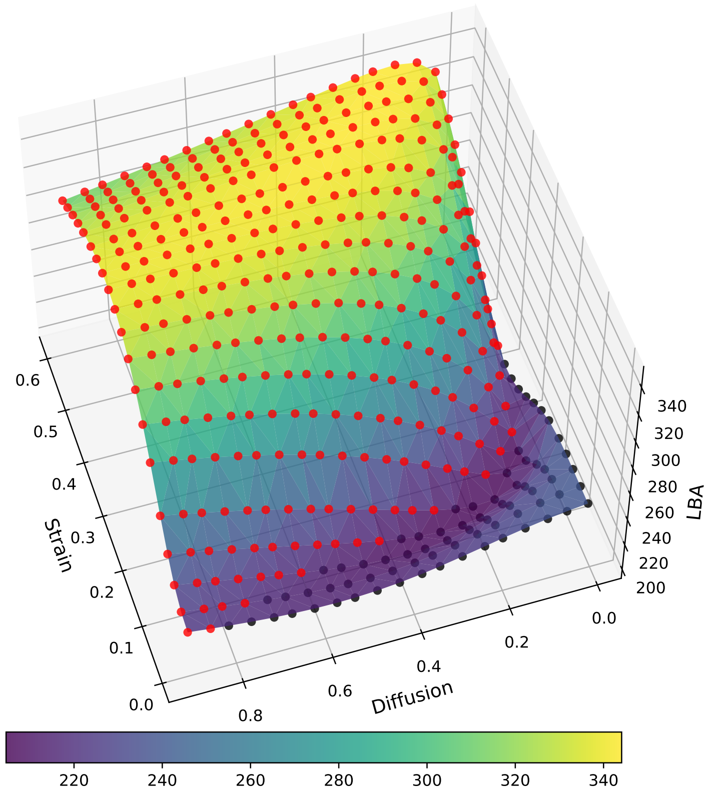

SUPPLEMENTARY FIGURE 6. LBA surface of the heterogeneous ensemble of Fig. 6 of main text. We add colored dots corresponding to each single simulation. The dot is black if the term in the consumer growth rate associated with second moments is zero or negative, that means: $\frac{\langle p_r' p_b' \rangle}{(\langle p_r \rangle + k)^2} \leq \frac{\langle p_b \rangle \langle p_r'^2 \rangle}{(\langle p_r \rangle + k)^3}$. The dot is red if the term in the consumer growth rate associated with second moments is positive, that means: $\frac{\langle p_r' p_b' \rangle}{(\langle p_r \rangle + k)^2} > \frac{\langle p_b \rangle \langle p_r'^2 \rangle}{(\langle p_r \rangle + k)^3}$.

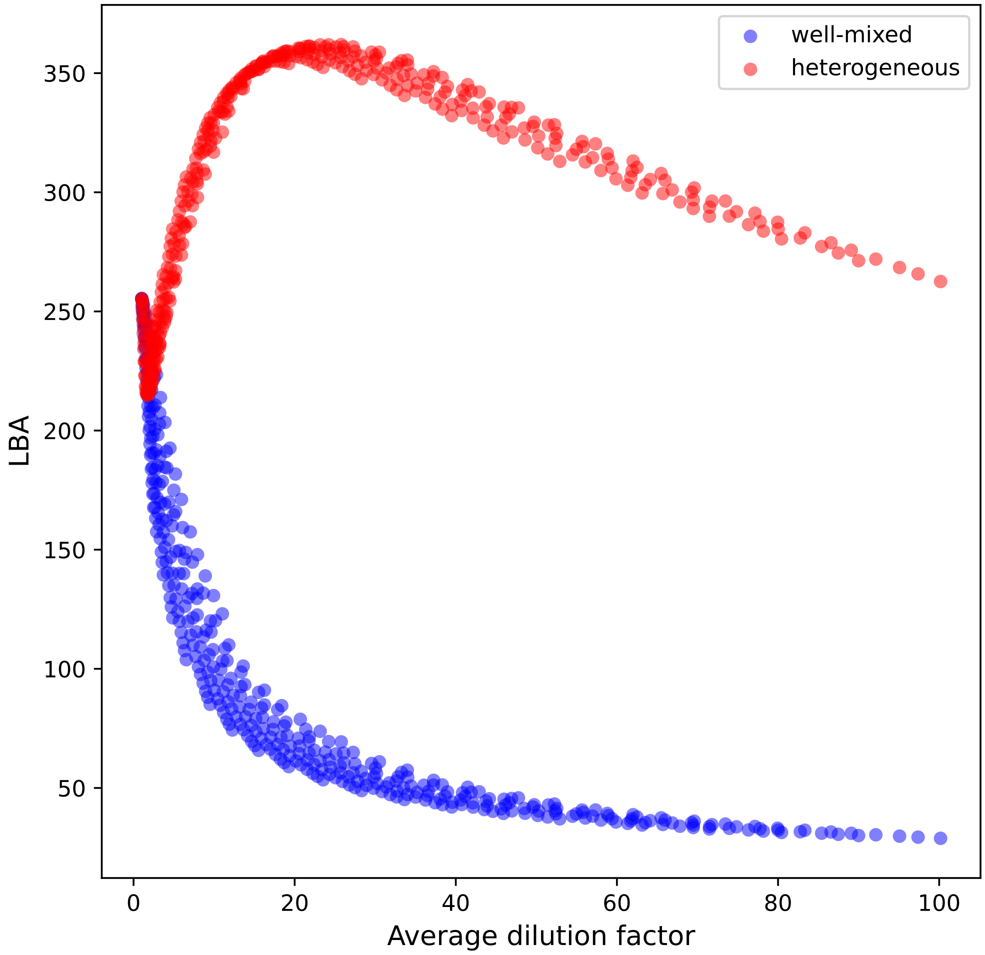

SUPPLEMENTARY FIGURE 7. LBA versus dilution scatter plot ensembles of heterogeneous (red) and well-mixed (blue) patches as in Fig. 7 of main text but setting the surrounding tracer concentrations to zero, that means: $\langle s_r \rangle = \langle s_b \rangle = 0$. Each dot corresponds to a single simulation.

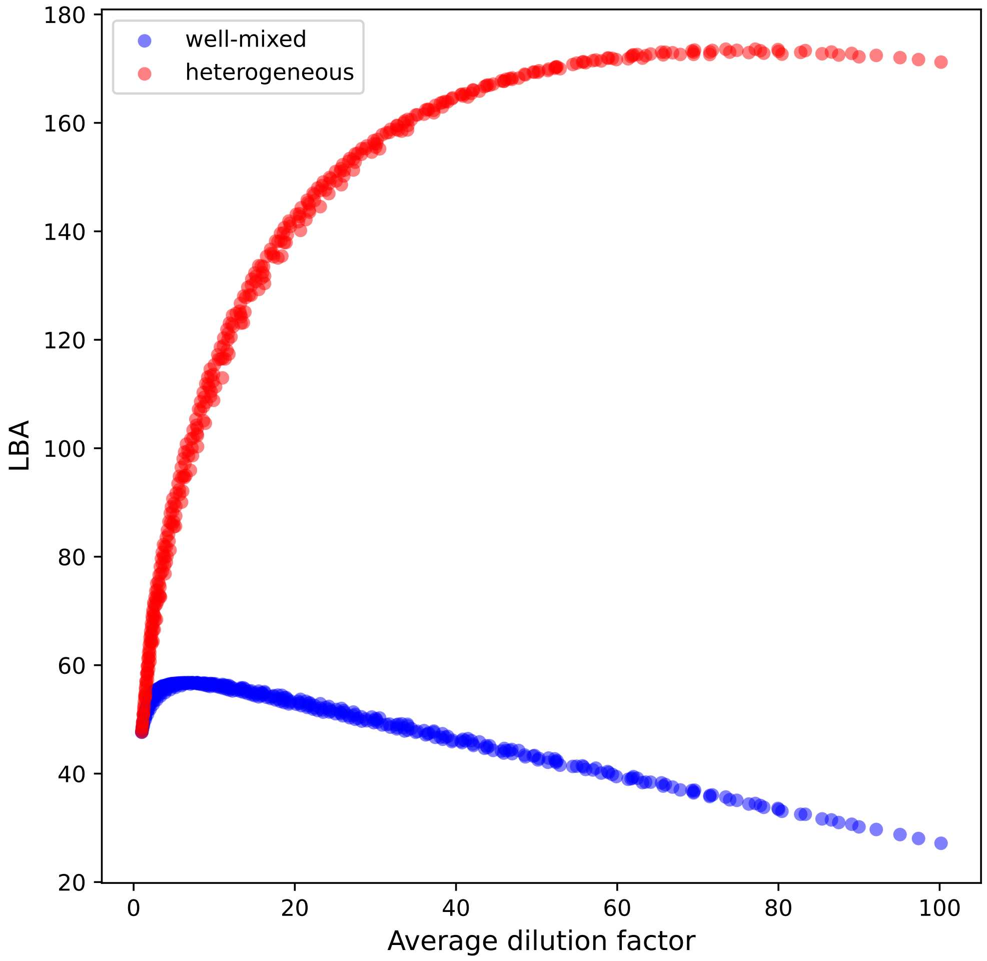

SUPPLEMENTARY FIGURE 8. LBA versus dilution scatter plot ensembles of heterogeneous (red) and well-mixed (blue) patches as in Fig. 7 of main text but using a quadratic mortality term in Eq. (10). Each dot corresponds to a single simulation.
